# Implications of the Adiponectin System in Non-Small Cell Lung Cancer Patients: A Case-Control Study

**DOI:** 10.3390/biom10060926

**Published:** 2020-06-18

**Authors:** Ersilia Nigro, Fabio Perrotta, Maria Ludovica Monaco, Rita Polito, Pia Clara Pafundi, Maria Gabriella Matera, Aurora Daniele, Andrea Bianco

**Affiliations:** 1 Dipartimento di Scienze e Tecnologie Ambientali, Biologiche, Farmaceutiche, Università della Campania “Luigi Vanvitelli”, Via G. Vivaldi 42, 81100 Caserta, Italy; nigro@ceinge.unina.it (E.N.); RITA.POLITO@unicampania.it (R.P.); 2 CEINGE—Biotecnologie Avanzate, Via G. Salvatore 486, 80131 Napoli, Italy; ml.monaco@unicampania.it; 3 Department of Medicine and Health Sciences “V. Tiberio”, University of Molise, 86100 Campobasso, Italy; fabio.perrotta@unimol.it; 4 Department of Advanced Medical and Surgical Sciences, Università della Campania “Luigi Vanvitelli”, Piazza Miraglia, 80138 Naples, Italy; piaclara.pafundi@unicampania.it; 5 Unit of Pharmacology, Department of Experimental Medicine, University of Campania “Luigi Vanvitelli”, Via Santa Maria di Costantinopoli, 80138 Naples, Italy; mariagabriella.matera@unicampania.it; 6 Department of Translational Medical Sciences, University of Campania “L. Vanvitelli”, 80131 Naples, Italy; andrea.bianco@unicampania.it; 7 Monaldi Hospital, 80131 Naples, Italy

**Keywords:** lung cancer, NSCLC, adiponectin, HMW oligomers, adiponectin receptors

## Abstract

Alterations of adipose tissue occurring in obesity have been recognized as a major risk factor for several cancers. The relationship between adipose tissue and lung cancer, which is the main cancer-related cause of death worldwide, still requires investigation. Perturbations in the adipokine system are likely to interfere with inter-organ crosstalk in lung cancer, which may influence the lung tumor microenvironment. Adiponectin (Acrp30) expression is deregulated in several cancer types. Acrp30 circulates as oligomers with a Low (LMW), Medium (MMW), and High Molecular Weight (HMW), with the latter mediating the main biological effects. Acrp30 acts through AdipoR1 and AdipoR2 receptors. T-cadherin has been described as a non-signaling receptor. This study’s aim was to investigate the regulation of serum Acrp30 and its receptors in sample tissue from non-small cell lung cancer (NSCLC) patients. We recruited 72 NSCLC patients and 60 healthy controls, whom we evaluated in terms of their Acpr30 levels and oligomeric profile. In addition, the expression of AdipoRs in tissues from lung cancer specimens was also measured and compared to coupled healthy lung samples. Our findings show a significant reduction of total Acrp30 levels in NSCLC patients compared to normal subjects, with a specific down-regulation of HMW oligomers. Acrp30 expression was lower in lung adenocarcinoma than other subtypes, regardless of other factors. A significantly higher expression of AdipoR1 was observed, while no differences in R2 and a lower expression of T-cadherin were found in lung cancer specimens compared to normal healthy lung tissues. Involvement of the Acrp30 system in lung cancer may provide new insight into the interaction between adipose tissue and lung and sheds light on its potential ability to influence the lung tumor microenvironment.

## 1. Introduction

Lung cancer is the main cancer-related cause of death in developed countries, with an unsatisfactory five-year survival rate, ranging from 10 to 15% [1,2]. Non-small cell lung cancer (NSCLC) accounts for 85–90% of patients and can be further stratified, based on histology, into adenocarcinoma (AD), squamous cell carcinoma (SC), large cell carcinoma (LCC), and “others” [1,3]. Despite substantial advances in our understanding of the molecular basis of lung cancer [4,5,6,7,8,9], ongoing research on driver genes, mechanisms of immune evasion, and the tumor microenvironment, which also triggers crosstalk phenomena between organs/tissues, is expected to improve both early disease detection and survival [10]. Data from genome-wide (GWAS) and transcriptome-wide association studies (TWAS) in large lung cancer cohorts have documented prominent heterogeneity in the genetic susceptibility across lung cancer histological subtypes, possibly reflecting different underlying oncogenic molecular drivers [11,12,13]. Recently, obesity was recognized as a major risk factor linked to both the incidence and progression of several cancer types [14]. However, the molecular and cellular mechanisms by which adipose tissue affects both tumor initiation and progression have not yet been completely elucidated. Nevertheless, it is well-known that, beyond the adipose tissue volume, the presence of either inflammation/adipocyte hypertrophy or hypoxia reflects the metabolic and inflammatory status involved in the disruption of local and systemic physiological body homeostasis [15]. Adipocytes, through the production and secretion of different adipokines, while facilitating inter-organ crosstalk, indirectly affect the biology of tumor cells by regulating insulin resistance and inflammation [16,17].

Among other adipokines, adiponectin (Acrp30), in addition to insulin-sensitizing and anti-inflammatory functions, is downregulated in serum by different types of cancer [18]. Acrp30 is the most abundant adipokine secreted by adipose tissue, and circulates at high concentrations (5–30 μg/mL) as oligomers of different molecular weights:

- Low Molecular Weight (LMW);
- Medium Molecular Weight (MMW);
- High Molecular Weight (HMW) oligomers [19].

It has been widely reported that HMW oligomers mediate the main active biological effects of the protein [20]. Acrp30 acts through two signaling receptors—AdipoR1 and AdipoR2—which are widely expressed in several organs, tissues, and cell lines [21,22,23]. In addition, a third protein, known as T-cadherin, has been described as a receptor mainly expressed in the vascular system and specific for High and Medium Molecular Weight Acrp30 [24,25,26].

Several data sets support the hypothesis of both a direct and indirect role of Acrp30 as a regulatory mediator of different mechanisms underlying lung carcinogenesis [15,27]. From a molecular point of view, Acrp30 inhibits carcinogenesis by regulating both cell growth and inflammatory cytokine levels [7]. Moreover, Acrp30 serum levels have been found to be heterogeneously expressed in patients with cancer [28,29]. Altogether, these observations show that Acpr30 may have an important role in both the establishment and progression of lung cancer, inhibiting both processes.

In this study, we aimed to investigate differences in adiponectin serum levels in patients with NSCLC compared to healthy controls. Furthermore, we evaluated the AdipoRs expression of healthy lung and neoplastic tissue. Therefore, we analysed Acpr30 levels and their expression profile, particularly focusing on HMW forms. In addition, we also evaluated AdipoRs expression, both at mRNA and protein levels, in tissue specimens from a cohort of lung cancer patients.

## 2. Material and Methods

### 2.1. Subjects

A total of 72 unrelated subjects (46 M/26 F) with NSCLC were recruited from the Respiratory Diseases Unit of the Department of Translational Medical Sciences, University of Campania “Vanvitelli”, Italy and compared to 60 age-matched healthy controls recruited at CEINGE (Naples, Italy). All subjects aged >18 years with newly diagnosed NSCLC (stage I–IV) were included in the study. Patients with any previously diagnosed different types of cancer—other than non-melanoma skin cancer—were excluded. For both lung cancer patients and healthy controls, blood samples were collected after a 12-h overnight fasting period and centrifuged to collect serum. Serum aliquots were immediately frozen in liquid nitrogen and stored at −80 °C. The Body Mass Index (BMI) was calculated as previously reported [30].

Lung tissues from NSCLC specimens were collected from the first twenty consecutive patients. The study was approved by a local Ethics Committee and conducted in accordance with the 1976 Declaration of Helsinki and its later amendments. Written informed consent was obtained from all participants.

### 2.2. Anthropometric and Biochemical Measurements

For all participants, the total cholesterol, high-density lipoprotein (HDL), low-density lipoprotein (LDL), triglyceride, fasting glucose, aspartate transaminase (AST), alanine transaminase (ALT), and gamma glutamyl transferase (GGT) levels were collected. The serum total Acrp30 concentration was measured in all individuals in triplicate by an enzyme-linked immunosorbent assay (ELISA) using a polyclonal antibody produced in-house versus a human Acrp30 amino acid fragment (H2N-ETTTQGPGVLLPLPKG-COOH), as previously described [30] . Each serum sample was tested three times in triplicate.

### 2.3. Western Blotting Analysis

Total serum proteins were quantified by Bradford’s method (Bio-Rad, Hercules, CA, USA); 10 µg was treated with 1X Laemmli buffer, heated to 95 °C for 10 min, and loaded on 10% SDS-PAGE gel, as previously described [31] Blots were developed by ECL (Amersham Biosciences, Piscataway, NJ, USA) with the use of Kodak BioMax Light film, digitalized with a scanner (1200 dpi), and analyzed by densitometry with the ImageJ software (Available online: http://rsbweb.nih.gov.ij/). Each sample was tested three times in duplicate.

Lung specimens were obtained from the neoplastic tissue and normal lung parenchyma of 20 NSCLC patients after being lysed and homogenized in RIPA buffer (Sigma-Aldrich, St. Louis, MO, USA). The lysate proteins were quantified by the Bradford method and 25 µg of proteins was dissolved in 1X Laemmli buffer and separated using 10% SDS-PAGE gel, as previously described [30]. Incubation with AdipoR1, AdipoR2 (Santa Cruz Biotechnology, Dallas, TX, USA), T-cadherin (Abcam, Cambridge, UK), and Glyceraldehyde-3-Phosphate Dehydrogenase (GAPDH) primary antibodies (Sigma-Aldrich, St. Louis, MO, USA) was performed according to the manufacturer’s instructions. The blots were developed by ECL (Amersham Biosciences, Piscataway, NJ, USA) and Kodak BioMax Light film, digitalized with a scanner (1.200 dpi), and analyzed by densitometry with the ImageJ software (available online: http://rsbweb.nih.gov.ij/).

### 2.4. RNA Extraction and Real-Time Quantitative PCR

Total RNA was isolated in both neoplastic and normal lung parenchyma using TRIzol (Invitrogen, CA). Real-time quantitative PCR was carried out for 40 cycles at a melting temperature of 95 °C for 15 s and an annealing temperature of 60 °C for 1 min. A dissociation curve was analyzed for each PCR experiment to assess either primer–dimer formation or contamination. Relative mRNA level quantifications of target genes were determined by the cycle threshold method with GAPDH as the housekeeping gene, and data were expressed as the expression relative to the housekeeping gene. AdipoR1, AdipoR2, CDH13, and GAPDH primers are available on request. The experiments were performed two times in triplicate.

### 2.5. Gel Filtration Analysis

Acrp30’s oligomeric distribution in serum samples was analyzed by gel filtration chromatography on a Superdex 200 10/300 GL column connected to a fast protein liquid chromatography system (Amersham Pharmacia Biotech, Upsala, Sweden). Specifically, about 1875 μg of total proteins contained in about three hundred microliters was fractionated at 0.5 mL/min using a 100 mmol/L PBS, pH 7.4 elution buffer. The column was calibrated using Ferritin (440 kDa), Aldolase (158 kDa), and Ovalbumin (44 kDa) (GE Heathcare, Little Chalfont, UK). Fractions (250 μL) were collected and the presence of Acrp30 oligomers in Fast protein liquid chromatography (FPLC) fractions was tested by both the ELISA assay and western blot analysis.

### 2.6. Statistical Analysis

Data are shown as either the median or range in the case of continuous variables or number and percentage for categorical variables. All continuous variables were previously tested for normality by the Kolmogorov Smirnov and the Shapiro Wilk goodness-of-fit tests. Differences between groups were analyzed by Fisher’s exact test or a Chi-square test for categorical variables. A non-parametric Mann–Whitney U test or Kruskal–Wallis test was performed to compare continuous variables.

All variables that were statistically significant in the univariate analysis were further tested for a potential independent association with the outcome of interest. A multivariable logistic regression model, with a stepwise selection method, was performed.

Finally, bivariate correlations were tested by the Spearman’s correlation coefficient. All tests were two-tailed and a *p*-value < 0.05 was considered statistically significant. Data were analyzed using SPSS Software, Version 24 (IBM, Armonk, NY, USA), and STATA 14.0 software (StataCorp. 2015. StataCorp LP, College Station, TX, USA).

## 3. Results

### 3.1. Baseline Features and Serum Levels of Total and HMW Acrp30

The anthropometric and biochemical characteristics of both NSCLC patients and age-matched controls are shown in Table 1. Higher levels of glucose and GGT were observed in patients compared to controls. The analysis of total serum Acpr30 levels revealed a significantly lower concentration in the NSCLC group (median 10.8 μg/mL vs. 15.5 μg/mL; *p* < 0.001). Moreover, the fasting glucose, ALT, AST, and GGT levels were significantly higher among NSCLC patients compared to among controls. In addition, a weak correlation between total Acrp30 and triglycerides in the NSCLC cancer patients group emerged (Spearman’s rho = 0.246; *p* = 0.037). The entire correlation coefficient analysis is reported in Table S1. A binary logistic regression, with stepwise method selection, was further performed to investigate the influence of adiponectin levels on NSCLC histology (adenocarcinoma versus other subtypes). Lower Acpr30 levels were documented in adenocarcinoma patients (*n* = 30, median 9.5 µg/mL (QR 8–10.7)) than in NSCLC with other subtypes (*n* = 40, median 12.4 µg/mL (IQR 10.5–14.6)). In the multivariable logistic regression analysis, adenocarcinoma emerged as independently associated with Acpr30 (OR 1.453, 95% CI 1.176–1.795; *p* = 0.001). Correlation coefficients for adiponectin with clinical, laboratory, and pathological characteristics in NSCLC patients are reported in Table 2. As presented in Table 2, only histologic classification shows a significant positive linear correlation with serum Acrp30 from adenocarcinoma patients exhibiting lower Acpr30 levels compared with other subtypes. No other linear correlation appears clear from our data, though the slightly inverse correlation with cholesterol and fasting glucose is of interest and should be investigated further.

**Figure 1:**
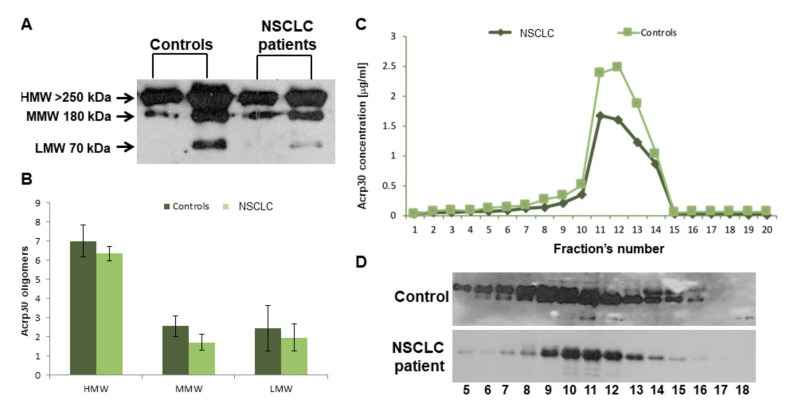
Western Blotting (WB) and FPLC analysis of adiponectin oligomers. (**A**) Western blot of the three Acpr30 oligomers High Molecular Weight (HMW), Medium Molecular Weight (MMW), and Low Molecular Weight (LMW)) in two controls and two lung cancer patients. (**B**) Pixel quantization of adiponectin oligomers of all analyzed controls (*n* = 60) and lung cancer patients (*n* = 72). (**C**) Each fraction’s aliquot obtained from FPLC analysis was subjected to ELISA. The values are reported as the mean of the absorbance ± SD. (**D**) Western blot analysis of each fraction obtained from FPLC (further details are given in the methods section).

The next step was to characterize Acrp30’s oligomeric distribution by western blot analysis (Figure 1A,B) and FPLC chromatography (Figure 1C,D). Both analyses showed reduced levels of the three Acrp30 oligomers in lung cancer patients, particularly with regard to HMW, the most bioactive oligomers.

### 3.2. AdipoR1, AdipoR2, and T-Cadherin Expression in Lung Tissues

**Figure 2:**
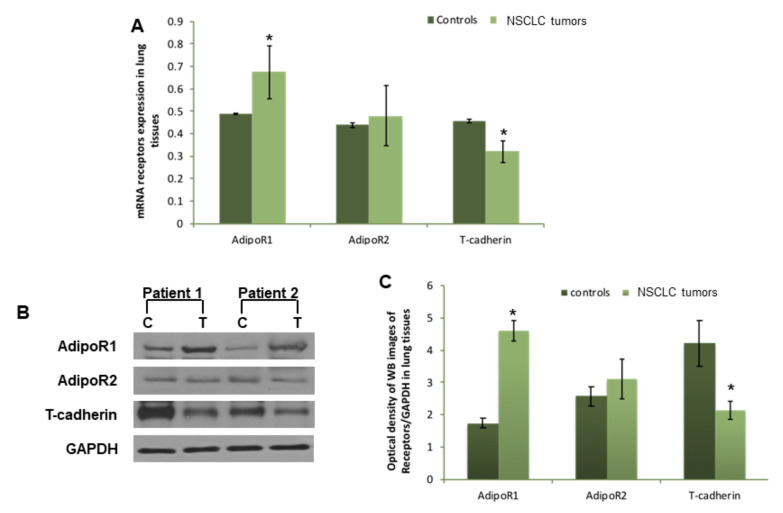
Different modulation of AdipoR1, AdipoR2, and T-cadherin expression in cancerous lung tissues compared to non-cancerous tissues. (**A**) Real-time PCR analysis of AdipoR1, AdipoR2, and T-cadherin relative to GAPDH expression in lung non-cancerous tissues and cancerous tissues (data expressed as the mean of 2^−ΔCt^). (**B**) One representative western blot image of AdipoR1, AdipoR2, T-cadherin, and GAPDH in lung non-cancerous tissues compared to cancerous tissues; (**C**) pixel quantization representation of AdipoR1, AdipoR2, and T-cadherin in 20 lung non-cancerous tissues and cancerous tissues. * *p* < 0.05 (Student *t*-test). For further details, see the materials and methods section.

Considering the difference in both Acrp30’s serum levels and oligomeric distribution, we successively considered the expression of the three Acrp30 receptors in lung specimens obtained from patients undergoing surgery (Figure 2A–C). Therefore, we analyzed AdipoR1, AdipoR2, and T-cadherin at both an mRNA and protein level by real-time PCR and western blot analysis. Both investigations showed a significantly higher expression of AdipoR1 at both an mRNA (Figure 2A) and protein level (Figure 2B,C) in neoplastic samples compared to healthy parenchyma. Conversely, T-cadherin was significantly down-regulated in cancerous tissue compared to non-cancerous tissue at both an mRNA (Figure 2A) and protein level (Figure 2C). Finally, AdipoR2 was only slightly up-regulated in both neoplastic tissue and normal lung parenchyma (Figure 2B,C).

## 4. Discussion

In this study, the adiponectin system has been explored by the detection of both the serum level of Acrp30 and its oligomeric distribution and the cell adipo-receptor in tissues from lung cancer patients. We have demonstrated a statistically significant reduction of total Acrp30 levels in cancer patients compared to healthy controls. Interestingly, patients with an adenocarcinoma subtype expressed lower levels when compared to other subtypes. Furthermore, the Acrp30 oligomers’ profile underlined a lower expression of all Acrp30 oligomers, in particular of HMW oligomers, which are the most biologically active. Finally, we documented that AdipoR1 expression is significantly up-regulated, while T-Cadherin is down-regulated, in NSCLC tissues.

A number of soluble mediators, along with cells, signaling molecules, and the extracellular matrix (altogether constituting the tumor microenvironment), play a crucial role in both promoting or not-promoting carcinogenesis and conferring resistance to therapy [32]. Cytokines and hormones produced by adipose tissue are both directly and indirectly involved in the creation of the tumor microenvironment, as they take part in cellular processes such as proliferation, apoptosis, and inflammation [32,33]. Among adipokines, growing interest has focused on Acpr30 and its HMW oligomers, since their levels are altered in several lung pathological conditions, such as Chronic Obstructive Pulmonary Disease (COPD) and asthma [34,35].

With regard to receptors for adiponectin, we observed a different trend for each specific receptor. Indeed, AdipoR1 expression was significantly higher in lung cancer specimens than normal healthy lung tissues. Finally, no differences were observed in R2, even when the expression of T-cadherin was lower.

Despite the strong evidence for Acrp30’s crucial role in lung physio-pathological conditions, controversial results on Acpr30 levels in lung cancer patients have been reported. In accordance with our results, Petridou et al. did not observe any significant difference in Acpr30 concentrations in patients with lung cancer compared to controls, despite significantly lower levels in patients with an advanced stage of the disease [36]. On the contrary, Karapanagiotou et al. reported no significant difference in Acpr30 serum levels in advanced NSCLC patients [37]. Furthermore, Kerenidi et al. demonstrated a significant increase in serum Acpr30 levels in lung cancer patients [38], but, contrary to our data, did not find any correlation with clinical data. Although the sample size between the two studies is comparable, several factors might explain this inconsistency, such as the age, sex, BMI, and tumor subtypes of the studied population. Furthermore, our findings suggest a potential difference in histology subtypes, documenting lower serum Acrp30 in patients with adenocarcinoma. These results have never been previously reported in NSCLC. Conversely, at other cancer sites, a correlation between serum adiponectin and specific cancer subtypes has been suggested. Wang et al. showed that lower Acpr30 levels were independently associated with clear cell renal carcinoma (ccRCC) when compared to non ccRCC (*p* = 0.004) [39]. The down-regulation of Acrp30 levels might represent a relevant factor directly regulating tumor growth. Indeed, circulating hormones and growth factors influence tumorigenesis, modifying the stromal microenvironment [40].

Regarding AdipoRs expression, Petridou et al. demonstrated a higher expression of both AdipoR1 and 2 in cancer lung tissues [36]. The third Acpr30 receptor, T-cadherin, most likely acts as a co-receptor with the more classic AdipoR-1 and -2 in the binding of hexameric and larger Acrp30 forms [23,41]. T-cadherin was discovered to be a unique “truncated” cadherin associated with the plasma membrane, though lacking cytoplasmic cytoplasm sequences [42]. It is likely that T-cadherin sequesters Acrp30, but also serves as an Acrp30 repository [43]. Therefore, T-cadherin not only regulates circulating and tissue-bound Acrp30 levels, but also competes with Adipo R1 and R2 receptors for Acrp30 binding and interferes with the coupling of both receptors to their downstream intracellular targets [42,43]. In this scenario, the decreased expression of T-cadherin observed in lung cancer tissues supports the role of this receptor in Acrp30 regulation. Previous studies have suggested that almost all Acrp30 metabolic effects are conferred by AdipoR1 and R2 receptors [22]. Bag and Anbarasu analyzed functional gene interactions of Acrp30 and observed that, in contrast to AdipoR1 and AdipoR2 (mostly involved in glucose and lipid metabolic processes), the T-cadherin gene participates in the cell adhesion process [44]. In tumors, T-cadherin/CDH13 is often silenced in cancer cells, but up-regulated in tumor vasculature; its T-cadherin-dependent accumulation in a tumoral microenvironment may favor both neoangiogenesis and tumor growth [45]. Therefore, T-cadherin down-regulation seems to play an important role in inducing malignant phenotypic change and tumorigenicity in lung cancer. One of the limitations of the study is the lack of information about T-cadherin expression specifically in vasculature and therefore, Acrp30 retention in the tumor microenvironment. In addition, the limited number of lung specimens represents another limitation, because it did not allow any inferential analysis of AdipoRs in correlation with tumor characteristics.

We can speculate that a bidirectional regulation could exist between tumor microenvironment (TME) and adipose tissue. On one hand, the observed down-regulation of T-cadherin (indicated as the storage Acrp30 receptor) in tumor tissues has been reported to be associated with an up-regulation of T-cadherin in the vasculature that could partially determine a reduction in Acrp30 production by the local infiltrating adipose tissue; this decrease of local Acrp30 in turn determines AdipoR1 up-regulation (in this regard, TME affects adiponectin production and function). On the other hand, Acrp30 levels may be regulated by other factors, such as pro-inflammatory cytokines acting on adipose tissue; low levels of Acrp30 affect the expression levels of its receptors in lung tissues, which may have implications in TME regulation, also through the down-regulation of T-cadherin and up-regulation of AdipoR1. Whilst our data do not give evidence about mechanisms determining adiponectin down-regulation, they suggest that nearby and distant adipose tissue may participate in TME through several mechanisms.

**Figure 3:**
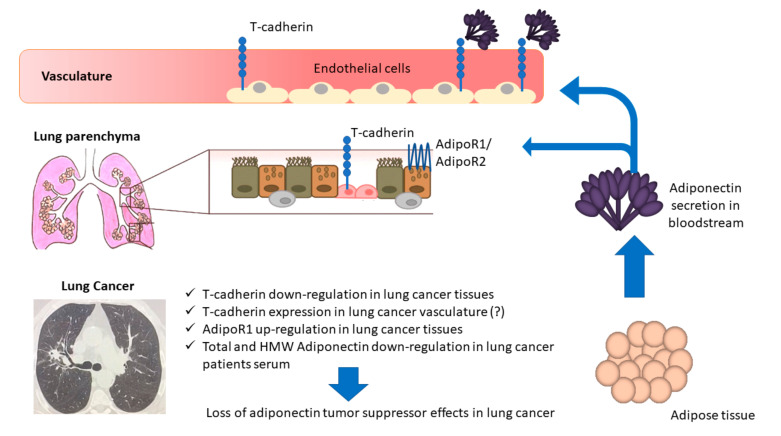
The down-regulation of T-cadherin in lung cancer tissues has been reported to be associated with an up-regulation of T-cadherin in the vasculature that could partially determine a reduction in Acrp30 production by the local infiltrating adipose tissue; this decrease of local Acrp30 in turn determines AdipoR1 up-regulation. On the other hand, Acrp30 levels may be regulated by other factors, such as pro-inflammatory cytokines acting on adipose tissue; low levels of Acrp30 affect the expression levels of its receptors in lung tissues, which may have implications in TME regulation, also through the down-regulation of T-cadherin and up-regulation of AdipoR1.

Our data support the concept that an alteration in adipokine homeostasis may modulate processes involved in cancerogenesis. Figure 3 schematically represents the findings of the present paper. Targeting the Acrp30 axis through the modulation of the hormone and related receptors induced by antidiabetic, antihypertensive, or immunomodulatory agents is under evaluation in early phase investigations. Evidence from studies of thiazolidinediones, fenofibrate, renin-angiotensin inhibitors, and mineralocorticoid receptor blockers have documented positive correlations with adiponectin serum level expression. In addition, as adipokines homeostasis appears to be modulated by physical training and pulmonary rehabilitation, this should also be explored in NSCLC cohorts [15,26,46,47,48,49].

## 5. Conclusions

Investigating the complex interaction between the Acrp30 system and lung cancer may provide new insight into the understanding of crosstalk between organs that interfere with tumor growth. Perturbations in the Acrp30 system, as we have demonstrated in lung cancer patients, may result in the modulation of anti-proliferative and anti-inflammatory effects induced by Acrp30, which might offer novel therapeutic options for patients with NSCLC. This may lead to further defining the real contribution of adipose tissue to both cancer development and progression.

Larger population studies are required to better establish the functional effects of Acrp30 down-regulation in lung cancer and to provide new insights into the implications for organ crosstalk on TME.

## Figures and Tables

**Table 1:** Anthropometric, clinical, and biochemical features in controls and lung cancer patients.

Parameters	NSCLC Patients (*n* = 72)	Controls (*n* = 60)	*p*
Sex, *n* (%)			0.300
*M*	46 (63.9)	33 (55)	
*F*	26 (36.1)	27 (45)	
Age (yrs.), median [IQR]	65 [58.5–70.5]	63 [51–76.8]	0.757
Weight (kg), median [IQR]	70 [64–79.5]	70.5 [64.3–81.8]	0.626
BMI (kg/m^2^), median [IQR]	25.3 [22.4–26.6]	24.9 [23.7–25.7]	0.696
Histology, n (%)			n.a.
*Adenocarcinoma*	32 (44.4)	-	
*Squamous Cell Carcinoma*	27 (37.5)	-	
Other *	13 (18.1)	-	
Stage, n (%)		-	
1/2	22 (30.6)		
3/4	50 (69.4)	-	
Performance status (ECOG), *n* (%)		-	
0/1	48 (66.7)		
2/3/4	24 (33.3)	-	
Lung Resection, *n* (%)	20 (27.8)	-	n.a.
Brain Metastases, *n* (%)	12 (16.6)	-	n.a.
Total Cholesterol (mg/dL), median [IQR]	180 (165–195)	179 (54.3–194.8)	0.874
Triglycerides (mg/dL), median [IQR]	110 (88.3–135.8)	98.5 (69–133.8)	0.167
Fasting Glucose (mg/dL), median [IQR]	99 (89.3–108.8)	89 (80.8–98.3)	< 0.001
AST, median [IQR]	20 (17–23.8)	17.5 (15–21.8)	0.039
ALT, median [IQR]	21 (17.3–27.8)	14 (11–22)	< 0.001
GGT, median [IQR]	30.5 (21.3–41)	16 (11–26.3)	< 0.001
Acpr30 (μg/mL), median [IQR]	10.8 (9.3–13.7)	15.5 (12.6–19)	< 0.001

Data are presented as the median and interquartile range (IQR). * Large cell carcinoma, adeno-squamous, and not otherwise specified. Abbreviations: IQR—interquartile range; M—male; F—female; BMI—Body Mass Index; ALT—alanine transaminase; AST—aspartate transaminase; GGT—gamma glutamyl transpeptidase; n.a. – not applicable; - missing.

**Table 2:** Linear Regression Model of serum total adiponectin in NSCLC Patient with respect to clinicopathologic features.

Parameters	Coefficient	95%	CI	*p*
Sex (M/F)	0.132	- 0.700	2.561	0.258
Age (years)	0.141	- 0.036	0.151	0.223
Stage (1/2 or 3/4)	0.010	- 1.849	2.000	0.938
Histology (adenocarcinoma, SCC or Other*)	0.347	0.566	2.606	0.003
BMI (kg/m^2^)	0.011	- 0.340	0.370	0.933
Cholesterol (mg/dL)	- 0.181	- 0.055	0.008	0.147
Triglycerides (mg/dL)	0.225	0.000	0.047	0.048
Fasting Glucose (mg/dL)	- 0.086	- 0.041	0.019	0.462
AST (U/L)	- 0.020	- 0.124	0.105	0.870
ALT (U/L)	0.019	- 0.072	0.083	0.886
GGT (U/L)	0.135	- 0.015	0.062	0.231

* Other: Large cell carcinoma, adeno-squamous, and not otherwise specified. Abbreviations: SCC—squamous cell carcinoma; M—male; F—female; BMI—Body Mass Index; ALT—alanine transaminase; AST—aspartate transaminase; GGT—gamma glutamyl transpeptidase.

## Supplementary Materials

The following are available online at https://www.mdpi.com/2218-273X/10/6/926/s1, Table S1: Correlation between Adiponectin levels and clinical and laboratoristic parameters.
