# Supplementary material for: Implications of the Adiponectin System in Non-Small Cell Lung Cancer Patients: A Case-Control Study
Source: Biomolecules. 2020 Jun 18;10(6):926. doi: 10.3390/biom10060926 (PMC7356727; doi:10.3390/biom10060926)
Supplement: Supplementary file 1 [file biomolecules-10-00926-s001.pdf]

Table S1: Correlation between Adiponectin levels and clinical and laboratoristic parameters

Parameters			
**Age**	Spearman's rho	0.214	
	p-value	0.078	
**Stage**	Spearman's rho	-0.072	
	p-value	0.549	
**Weight**	Spearman's rho	-0.038	
	p-value	0.751	
**BMI**	Spearman's rho	-0.008	
	p-value	0.944	
**Cholesterol**	Spearman's rho	0.011	
	p-value	0.928	
**Triglycerides**	Spearman's rho	0.246	*
	p-value	0.037	
**Glucose**	Spearman's rho	-0.055	
	p-value	0.646	
**AST**	Spearman's rho	-0.101	
	p-value	0.400	
**ALT**	Spearman's rho	0.066	
	p-value	0.579	
**GGT**	Spearman's rho	0.194	
	p-value	0.102	
